# The impact of problematic social media use on adolescent subjective well-being: insights from machine learning

**DOI:** 10.3389/fpsyt.2026.1764197

**Published:** 2026-07-20

**Authors:** Yuan Tian

**Affiliations:** School of Journalism & Communication, Southwest University of Political Science and Law, Chongqing, China

**Keywords:** adolescents, machine learning, multivariate logistic regression, problematic social media use, subjective well-being

## Abstract

Higher subjective well - being (SWB) among adolescents predicts higher educational achievements, work capabilities, and job satisfaction. Problematic social media use may adversely affect adolescents’ SWB. This study aims to identify key predictors of adolescents’ SWB by combining machine learning with multiple logistic regression methods, and to examine the relationship between problematic social media use and SWB. This study was based on data from the 2017/2018 Health Behaviour in School-aged Children (HBSC) survey. The data included the situations of problematic social media use of 187,090 adolescents aged between 10 and 16. The XGBoost and multivariate logistic regression models were used to explore the impacts of problematic social media use on adolescents’ SWB. With the exception of the time reduction failure dimension, all other dimensions of problematic social media use were negatively associated with SWB. Gain and SHapley Additive exPlanations (SHAP) analyses indicate that feeling escape, age, sex, and family conflict are key predictors of SWB. They also suggest that there may be complex interaction patterns among these variables. Specifically, feeling escape and family conflict account for 49.26% of the total variance, while age and sex account for 25.5%. Multivariate logistic regression results indicate that feeling escape, family conflict, increasing age, and female gender are all significantly associated with lower SWB. Therefore, future interventions should go beyond simply limiting social media usage time and focus on adolescents’ emotional regulation skills, the quality of family communication, and tailored support for older adolescents and girls.

## 1 Introduction

A growing body of research evidence suggests that adolescents’ Subjective Well-Being (SWB) is trending downward globally (1, 2). Discussions on the causes of this phenomenon have also intensified in numerous countries (3–6). SWB is an important indicator of an individual’s quality of life and psychological adjustment. It is generally understood as an individual’s subjective evaluation of their life circumstances, encompassing both cognitive assessments of life satisfaction and emotional experiences such as positive and negative emotions. Therefore, SWB is not merely an individual’s overall judgment of their life, but a multidimensional concept that incorporates both cognitive evaluations and emotional experiences (7, 8). SWB in adolescence is both an important outcome in its own right and a potential leverage point for promoting a healthy transition into adulthood (9). SWB in adolescence positively influences future productivity in adulthood. It predicts higher levels of education, job competence and job satisfaction in adulthood (10). The current findings suggest that interventions to promote SWB in adolescence may indeed be a viable way to promote a healthy transition into adulthood. Therefore, it is particularly important to identify the key factors that influence SWB in adolescents.

In recent years, social media has become an integral part of teenagers’ daily lives (11). Social media generally refers to internet- and mobile-based digital platforms that allow users to create, share, view, and interact with user-generated content, while also facilitating the establishment and maintenance of social relationships. Typical examples include social networking sites, instant messaging applications, video-sharing platforms, microblogging services, and online communities (12). Accordingly, social media use in the present study is defined as adolescents’ engagement with such platforms, including both active use, such as posting, commenting, messaging, and sharing, and passive use, such as browsing or viewing content. The use of social media also has a significant impact on SWB. Social media provides multiple functions for adolescents. It serves as a platform for adolescents to express themselves (13), a way to build and strengthen friendships (14), and a means to enhance social connections. In addition, it is an important channel for adolescents to access information, learn new skills (15) and engage in social issues (16). However, excessive, uncontrolled, or compulsive use can lead to problematic social media use and have adverse effects on mental health and SWB (17).

Problematic social media use has been defined as an excessive focus on social media and a strong drive that prompts individuals to log on or use social media platforms frequently, thereby investing too much time and energy, resulting in impairment of other social activities, school/work, relationships, and mental health and well-being (18). Research has shown that problematic social media use is strongly associated with lower SWB in adolescents (13, 19). And it is often accompanied by more serious psychological symptoms such as depression, anxiety, negative self-perceptions, and suicidal thoughts or behaviors (20, 21). The phenomenon has also fueled political calls for social media regulation (22).

Previous studies have confirmed a negative correlation between problematic social media use and adolescents’ SWB (19, 20, 23, 24). This study draws on the theory of compensatory internet use and social comparison theory as its theoretical framework.

The compensatory use theory of the Internet posits that individuals engage in online activities as a coping strategy aimed at alleviating negative emotions or life stress (25). Within this framework, social media use can be viewed as a form of emotional regulation, whereby adolescents turn to the online environment to escape dissatisfaction, stress, or psychological distress (26). When this pattern of use becomes excessive or uncontrolled, it can develop into problematic social media use. Although problematic social media use may provide some emotional relief in the short term, it is often accompanied by increased time spent online, avoidance of offline responsibilities (such as academic tasks and face-to-face interactions), and a growing dependence on the digital environment (27). Over time, this avoidance-based coping mechanism not only fails to fundamentally resolve emotional issues, but may actually exacerbate psychological distress, creating a self-perpetuating negative cycle (25). Compared to a mere increase in usage time, this emotion-avoidance-driven pattern of use exhibits stronger addictive properties and greater potential harm, thereby exerting a more profound negative impact on individuals’ overall SWB (28).

Complementing this view, social comparison theory posits that individuals evaluate themselves by comparing themselves to others, particularly in environments rich in social information (29). Social media platforms reinforce this process of emotional compensation by presenting adolescents with highly curated and idealized depictions of others’ lives (30). Such exposure may be associated with upward social comparison, which may in turn contribute to reduced self-esteem and increased feelings of inferiority. (31). Given that adolescents are at a critical stage of identity development and are particularly sensitive to peer evaluations, these comparative processes may have a significant impact on their SWB (32). At the same time, this effect may vary by age and sex. As adolescents grow older, their need for identity formation and peer acceptance intensifies, making them potentially more sensitive to social comparison information on social media (33). In terms of gender, girls typically exhibit greater sensitivity regarding appearance, self-presentation, and interpersonal evaluations. As a result, they are more susceptible to the influence of idealized online images. Boys, on the other hand, are more likely to be influenced by comparison processes related to social status, achievement, or peer recognition (34).

Based on these two theoretical perspectives, problematic social media use may influence adolescents’ SWB through a variety of interrelated pathways. These processes may also exacerbate interpersonal tensions, particularly within the family context, thereby further amplifying their negative impact on SWB (35). Therefore, there may be complex nonlinear relationships and multifactorial interactions between problematic social media use and adolescents’ SWB. However, existing empirical studies primarily rely on traditional regression methods, which tend to examine relationships between variables in isolation and struggle to fully capture these complex, nonlinear, and interactive mechanisms. Against this backdrop, machine learning methods offer an important complementary analytical framework by identifying higher-order interactions and latent patterns among predictor variables. (36).

Machine learning is a class of methods that use algorithms to identify patterns in data for classification or prediction. It is well-suited for handling data structures with large samples, multiple variables, and complex relationships (37).Machine learning methods demonstrate several key strengths in predicting adolescents’ SWB. First, machine learning has powerful information processing capabilities. Its algorithms are able to analyze large amounts of data simultaneously, overcoming the limitations of traditional statistical methods in terms of computational power and analytical flexibility. In this way, machine learning is able to recognize and predict hidden patterns in the data (38). Meanwhile, machine learning algorithms usually divide the dataset into a training set and a test set to ensure the reproducibility and generalization ability of the model. Independent validation of the test set ensures that the patterns extracted by the model are applicable to the entire dataset, thus improving the accuracy of the predictions (39). In addition, this study assessed the importance of each predictor variable by combining machine learning with methods such as SHapley Additive exPlanations (SHAP) analysis. This avoids the “black box” problem of traditional models and makes the model’s decision-making process more transparent and interpretable (40). This advantage allows us to identify and prioritize influential predictors to more effectively implement targeted interventions to improve SWB.

Although researchers have begun to use machine learning techniques to identify important predictors of adolescents’ SWB (41). However, the application of machine learning techniques in exploring the impact of problematic media use on adolescent SWB remains limited. The core goal of this study is to analyze Health Behaviour in School-aged Children (HBSC) data through machine learning methods to identify the problematic media use indicators most relevant to adolescent SWB. These indicators can provide a scientific basis for the Government, schools, parents and healthcare professionals to support them in implementing targeted interventions to enhance the SWB of adolescents and ultimately improve their overall physical and mental health.

Based on existing theoretical and empirical research, this study proposes the following research questions regarding the relationship between problematic social media use and SWB among adolescents. First, what are the associations between different dimensions of problematic social media use and adolescents’ SWB? Second, in the presence of complex nonlinear relationships, how can the relative importance of different factors in predicting SWB be ranked, and which variables emerge as key predictors? Third, are there complex interaction patterns between demographic characteristics and problematic social media use, and how are these interactions associated with variations in SWB in empirical data? By addressing these questions, this study aims to uncover the complex mechanisms linking social media use and adolescents’ SWB from a multidimensional perspective.

## 2 Methods

### 2.1 Study design and participants

This study employed a cross-sectional design utilizing data from HBSC 2017/2018 (https://hbsc.org/data/). The HBSC is the largest study on the health of children and adolescents, collecting comprehensive data on factors influencing the health and well-being of 11-, 13-, and 15-year-olds. Although the survey is designed to target these specific age groups, sampling is conducted at the school-grade level; consequently, participants may include students from adjacent age ranges. In the present study, participants were primarily aged between 10 and 16 years, and the term “adolescents” is used to refer to individuals within this range.

Data collection employs a standardized school-based questionnaire procedure. The survey is typically administered in a classroom setting by trained interviewers or school staff following standardized instructions. Participants complete anonymous questionnaires on an informed and voluntary basis to minimize social desirability bias and protect respondent privacy. Questionnaire content covers sociodemographic information, health behaviors, family and peer relationships, school life, mental health, and media use. Because the HBSC employs standardized survey instruments, sampling frameworks, and data management processes, this dataset exhibits a high degree of standardization and international comparability. The study adhered to the principles of the Declaration of Helsinki, ensuring the anonymity and confidentiality of all participants, and participation was voluntary. Lead institutions in all HBSC member countries are ethically licensed (42).

### 2.2 Measurement of variables

In this study, the variables used were assessed based on specific questions in the HBSC questionnaire. The variables cover both demographic characteristics and problematic social media use dimensions, and relevant information was collected through multiple choice items in the questionnaire. The demographic variables were age and sex. Participants were asked to indicate whether they were boys (coded 1) or girls (coded 2), as well as the month and year of their birth.

SWB was measured using a modified version of Cantril’s ladder (43). It provides participants with a picture of a ladder, with 10 at the top representing “Best possible life” and 1 at the bottom representing “Worst possible life”. Participants were asked to indicate where they felt they were on the ladder from 1 to 10. The Cantril Ladder has been widely used in adolescent well-being research and has demonstrated acceptable reliability and convergent validity across HBSC populations (44). Consistent with previous HBSC-based studies, the SWB variable was recoded into a binary classification (low: 0–5; high: 6–10). This threshold corresponds to the midpoint of the Cantril Ladder and has been widely used to distinguish between relatively low and relatively high levels of SWB among adolescents (45). Similar dichotomization procedures have been adopted in previous HBSC studies using the Cantril Ladder as the primary measure of adolescent SWB (46) Although the available SWB indicators in the present dataset yielded a Cronbach’s alpha of 0.42, this relatively low value may partly reflect the limited number of available items and the subsequent dichotomization of the outcome variable. Therefore, the Cantril Ladder was retained as the primary measure of SWB due to its extensive use in large-scale international surveys and established validity in adolescent populations.

Problematic (addiction-like) social media use was assessed through a validated nine-item social media disorder scale (47), which focuses on (1) can’t think of anything else; (2) spend more time; (3) felt bad; (4) failed to spend less time; (5) neglected other activities; (6) arguments because of use; (7) lied about amount; (8) escape from negative feelings; (9) conflict with family because of use. Participants responded “no” (coded 1) or “yes” (coded 2) to each item. These items have demonstrated strong psychometric properties, including high construct validity and good internal consistency (Cronbach alpha=0.9) (48). Based on the original structure of the HBSC dataset, the relevant variables were directly treated as binary indicators (yes/no). Participants answered each item with “no” (coded as 1) or “yes” (coded as 2). This scale was developed based on the criteria outlined in the Diagnostic and Statistical Manual of Mental Disorders (DSM-5) (49). It focuses on assessing the diagnostic presence of specific addiction-like symptoms rather than their frequency of occurrence. Furthermore, for the target sample of early adolescents (ages 10–16), the systematic binary response format designed by the HBSC significantly reduces cognitive load and recall bias, thereby enhancing the reliability of large-scale self-reported data.

### 2.3 Statistical analysis

This study used R software (Version 4.4.0) for statistical analysis. To systematically examine the impact of problematic social media use on adolescents’ SWB, this study employs both machine learning models and multivariate logistic regression models, which complement one another while focusing on different aspects.

Specifically, the XGBoost model is primarily used to capture complex nonlinear relationships and higher-order interactions among variables. It also employs feature importance ranking and SHAP analysis to identify key predictors influencing SWB. Although machine learning models have distinct advantages in classification tasks and handling nonlinear relationships, their results are relatively limited in terms of direct interpretability at the level of statistical inference. To address this limitation, this study further employs a multivariate logistic regression model. This provides estimates of the direction, strength, and statistical significance of the relationships between variables and SWB at the population level. Therefore, the regression analysis is not intended to replace or simply validate the machine learning model, but rather to verify whether key variables exhibit consistent directional effects within the framework of a parametric model. This enhances the interpretability and robustness of the research findings.

In terms of the specific analytical steps, we first use descriptive statistics to summarize the participants’ demographic characteristics. Second, the dataset in this study was divided into a training set accounting for 70% and a test set accounting for 30%, and the XGBoost model in the xgboost package was used for prediction. This model is particularly suitable for capturing the complex interactions between variables. Given the class imbalance in the data, this study employs a combined strategy of “weight adjustment and threshold optimization.” During the training phase, by setting the `scale_pos_weight` parameter, higher weights are assigned to underrepresented classes based on the sample distribution, thereby enhancing the model’s ability to learn features of these classes. During the prediction phase, the Youden Index is calculated based on the ROC curve to determine the optimal cutoff value, replacing the traditional 0.5 threshold, thereby achieving a better balance between sensitivity and specificity. Model performance is evaluated using metrics such as accuracy, sensitivity, and specificity. Then, with the help of the shapviz package, an important visual analysis was conducted on the key variables affecting the model prediction, and the importance of the key variables and their impacts on the model were examined in detail. Finally, since the variables related to problematic social media use were all binary variables, we further adopted a multivariate logistic regression model to test the impact of each variable on the SWB of adolescents. On this basis, the forestploter package was used to draw a forest plot and report the odds ratio (OR).

## 3 Results

Descriptive statistical analysis and chi-square test were performed for the variables of interest in this study. The results of the descriptive statistics in **Table 1** show that the mean sex score for these participants were 1.52, which indicates that there were approximately equal proportions of men and women in the sample. In addition, the mean SWB score in the sample was 0.87 ± 0.34. This result indicates that most participants reported high levels of SWB, but also reflects some inter-individual variability. The table also presents the *p*-values of the chi-square test for each variable, which were all less than 0.001, indicating a statistically significant relationship between these variables and SWB.

**Table 1:** Descriptive statistics and chi-square test of variables.

Variables	Label	Min	Max	Mean	Std. dev.	*p* value
Dependent Variable						
SWB	Subjective well-being	0	1	0.87	0.34	<0.001
Sociological and demographic factors						
Age	Age	10	16	13.12	1.64	<0.001
Sex	Sex	1	2	1.52	0.50	<0.001
Problematic Social Media Use						<0.001
Media obsession	Can’t think of anything else	1	2	1.21	0.41	<0.001
Media time increase	Spend more time	1	2	1.18	0.39	<0.001
Negative feelings	Felt bad	1	2	1.21	0.41	<0.001
Time reduction failure	Failed to spend less time	1	2	1.30	0.46	<0.001
Activity neglect	Neglected other activities	1	2	1.15	0.36	<0.001
Usage arguments	Arguments because of use	1	2	1.19	0.39	<0.001
Usage deception	Lied about amount	1	2	1.14	0.35	<0.001
Feeling escape	Escape from negative feelings	1	2	1.31	0.46	<0.001
Family conflict	Conflict with family because of use	1	2	1.14	0.35	<0.001

**Table 2** provides a descriptive statistical analysis of the study population, demonstrating the distribution of individual characteristics under different SWB categories. The study population was categorized into low and high SWB groups based on a range of demographic and problematic social media use factors. Out of a total of 187,090 participants, 51.98% were female and 95.14% were concentrated in the age group of 11 to 15 years. The low SWB group had higher proportions on all dimensions of problematic social media use than the high SWB group, which may indicate that problematic social media use is associated with lower SWB. Notably, the vast majority of adolescents (87.09%) exhibited high levels of SWB.

**Table 2:** Different individual characteristics across different SWB categories.

Variables	Characteristics	Low SWB		High SWB		Total	Percent
		n	%	n	%		
Age	10 years old	201	8.45%	2178	91.55%	2379	1.27%
	11 years old	4327	9.13%	43070	90.87%	47397	25.33%
	12 years old	1203	10.17%	10628	89.83%	11831	6.32%
	13 years old	7042	12.85%	47772	87.15%	54814	29.30%
	14 years old	1785	15.11%	10029	84.89%	11814	6.31%
	15 years old	8441	16.19%	43702	83.81%	52143	27.87%
	16 years old	1152	17.16%	5560	82.84%	6712	3.59%
Sex	Male	9246	10.29%	80597	89.71%	89843	48.02%
	Female	14905	15.33%	82342	84.67%	97247	51.98%
Media obsession	No	17109	11.65%	129780	88.35%	146889	78.51%
	Yes	7042	17.52%	33159	82.48%	40201	21.49%
Media time increase	No	17772	11.65%	134792	88.35%	152564	81.55%
	Yes	6379	18.48%	28147	81.52%	34526	18.45%
Negative feelings	No	16946	11.46%	130879	88.54%	147825	79.01%
	Yes	7205	18.35%	32060	81.65%	39265	20.99%
Time reduction failure	No	15345	11.79%	114775	88.21%	130120	69.55%
	Yes	8806	15.46%	48164	84.54%	56970	30.45%
Activity neglect	No	18839	11.88%	139683	88.12%	158522	84.73%
	Yes	5312	18.59%	23256	81.41%	28568	15.27%
Usage arguments	No	17501	11.49%	134825	88.51%	152326	81.42%
	Yes	6650	19.13%	28114	80.87%	34764	18.58%
Usage deception	No	19031	11.86%	141430	88.14%	160461	85.77%
	Yes	5120	19.23%	21509	80.77%	26629	14.23%
Feeling escape	No	12497	9.67%	116757	90.33%	129254	69.09%
	Yes	11654	20.15%	46182	79.85%	57836	30.91%
Family conflict	No	18534	11.55%	141978	88.45%	160512	85.79%
	Yes	5617	21.13%	20961	78.87%	26578	14.21%
Sum		24151	12.91%	162939	87.09%	187090	100.00%

**Table 3** presents the performance metrics of the XGBoost model, reflecting its overall performance in predicting SWB. Given the significant class imbalance in the dataset (Prevalence = 0.870), this study improved the model through weight adjustment and threshold optimization. The results show that the model’s accuracy is 66.5% (95% CI: 0.661–0.669), and the *p*-value from McNemar’s Test is less than 0.001, indicating that the model is statistically significant in distinguishing between positive and negative samples.

**Table 3:** The performance of the XGBoost model.

Items	Value
Accuracy	0.665
95% CI	(0.661, 0.669)
Mcnemar’s Test *p*-Value	<0.001
Sensitivity	0.684
Specificity	0.536
Pos Pred Value	0.908
Prevalence	0.870
Detection Rate	0.595
Detection Prevalence	0.656

In terms of classification performance, the model’s sensitivity was 0.684 and specificity was 0.536, indicating that after threshold optimization, the model achieved a certain balance in distinguishing between positive and negative samples. Furthermore, the positive predictive value was 0.908, indicating that the model exhibits high accuracy in identifying individuals with high SWB; the detection rate was 0.595, and the detection prevalence was 0.656. Overall, after accounting for class imbalance and making corresponding adjustments, the XGBoost model maintained a high ability to identify positive samples while also improving its ability to distinguish negative samples to some extent.

**Figure 1:**
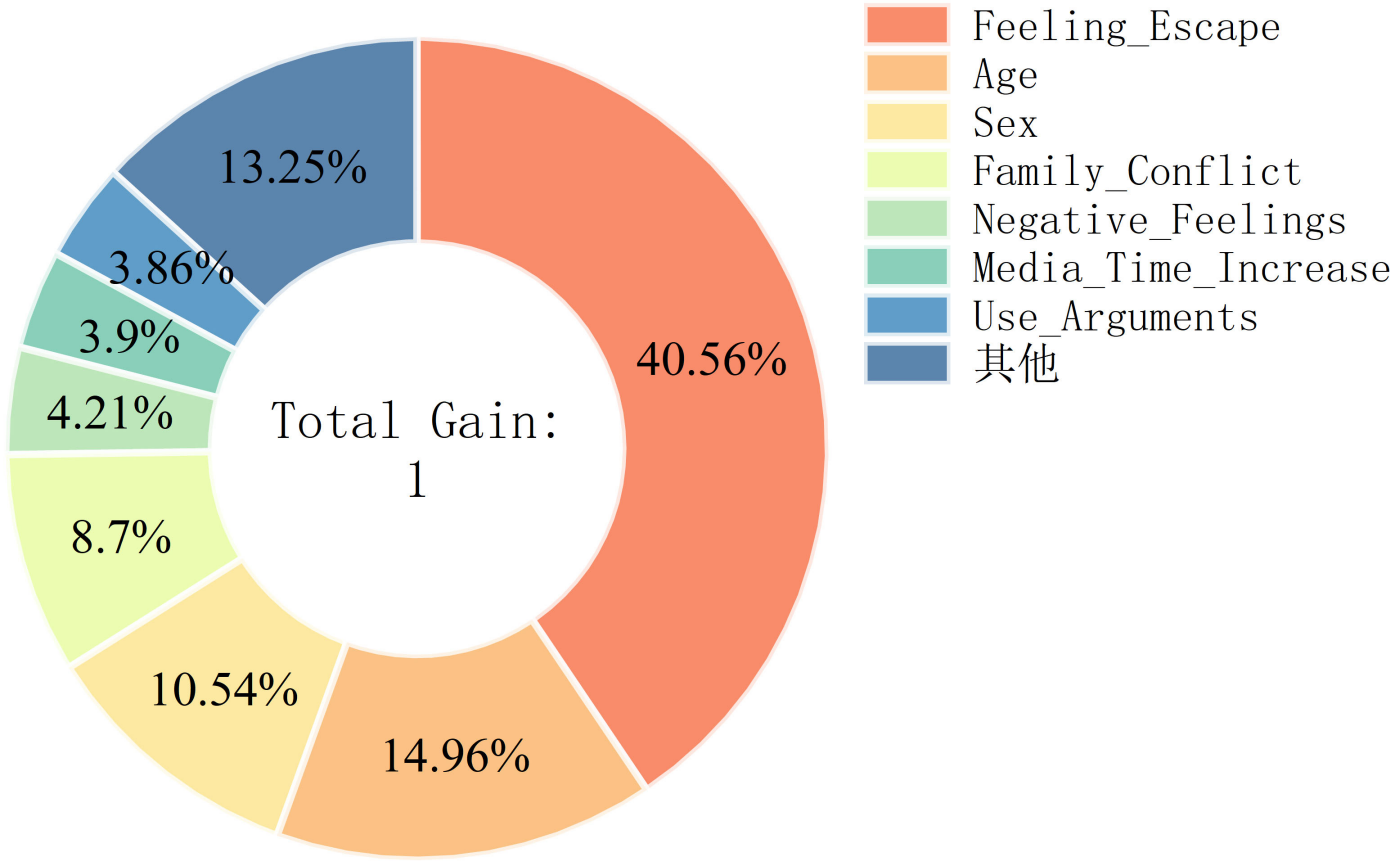
Importance ranking of factors influencing adolescent SWB in the XGBoost model.

For the XGBoost model, Gain was used to quantify the contribution of each feature to model accuracy during node splitting. Higher Gain values indicate that splitting on that feature can lead to greater model performance improvement. **Figure 1** illustrates the importance of each characteristic for adolescent SWB. Feeling escape occupies the most important place among these characteristics with a contribution of 40.56%. It was closely followed by age with a contribution of 14.96%. Sex and family conflict served as the third and fourth most influential factors. The characteristics of negative feelings, activity neglect, media obsession, and media time increase also had a predictive effect on the outcome. Other than that, the cumulative contribution of the other features is relatively low, suggesting that they play a lesser role in the model’s predictions.

**Figure 2:**
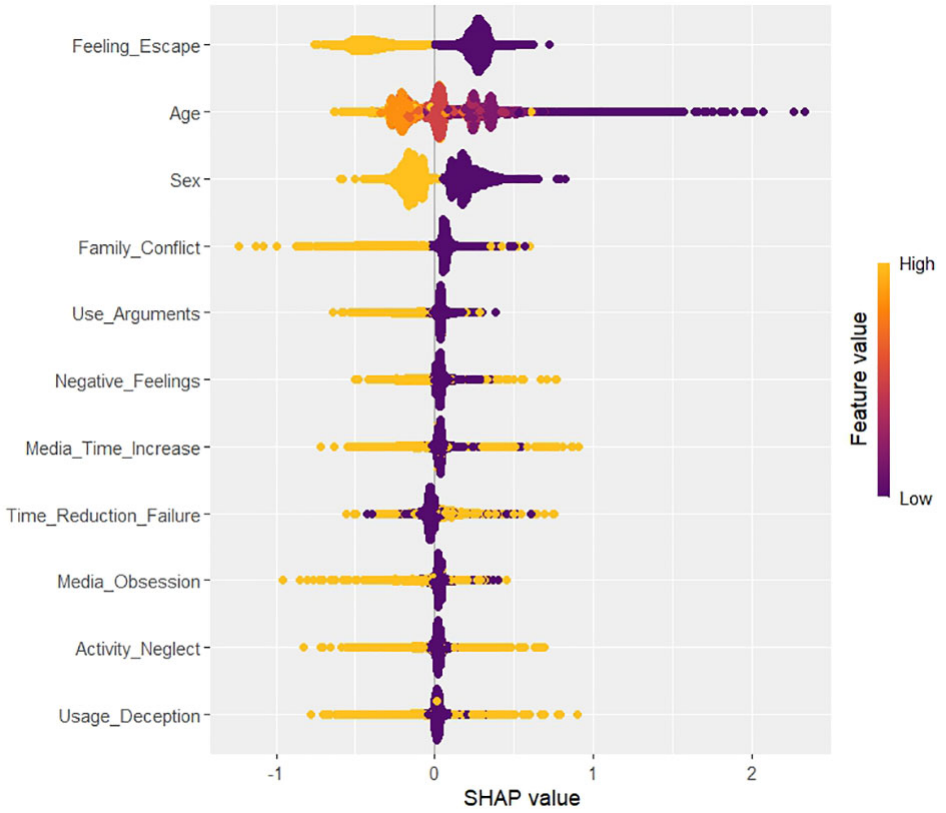
Beeswarm plot of factors influencing adolescent SWB in the XGBoost model.

**Figure 2** illustrates the density scatterplot of all 11 feature variables computed using the SHAP method. In **Figure 2**, each scatter represents the SHAP value of a sample, reflecting the feature’s contribution to an individual prediction. The scatter color from purple to yellow indicates the increasing value of the feature. The distribution of the scatters then indicates the overall direction and strength of the feature’s influence on the predictions. As can be seen in **Figure 2**, among these characteristics, feeling escape is the most important factor influencing the model’s prediction results. It presents a significant negative effect on the prediction results of SWB. A higher feeling escape, which means that adolescents will choose to use social media to escape negative emotions, will have a lower SHAP value and a lower predicted SWB. Age, sex and family conflict were the next most important characteristics. Older age (higher values) was associated with lower SHAP values and lower predicted SWB. Sex was female and also had lower SHAP values, resulting in lower predicted SWB. High values of family conflict usually corresponded to negative SHAP values, suggesting that family conflict resulting from the use of social media may decrease SWB. Other characteristics such as negative emotions and use of controversy were also shown to have an effect on the predicted outcomes, but probably to a lesser extent.

**Figure 3:**
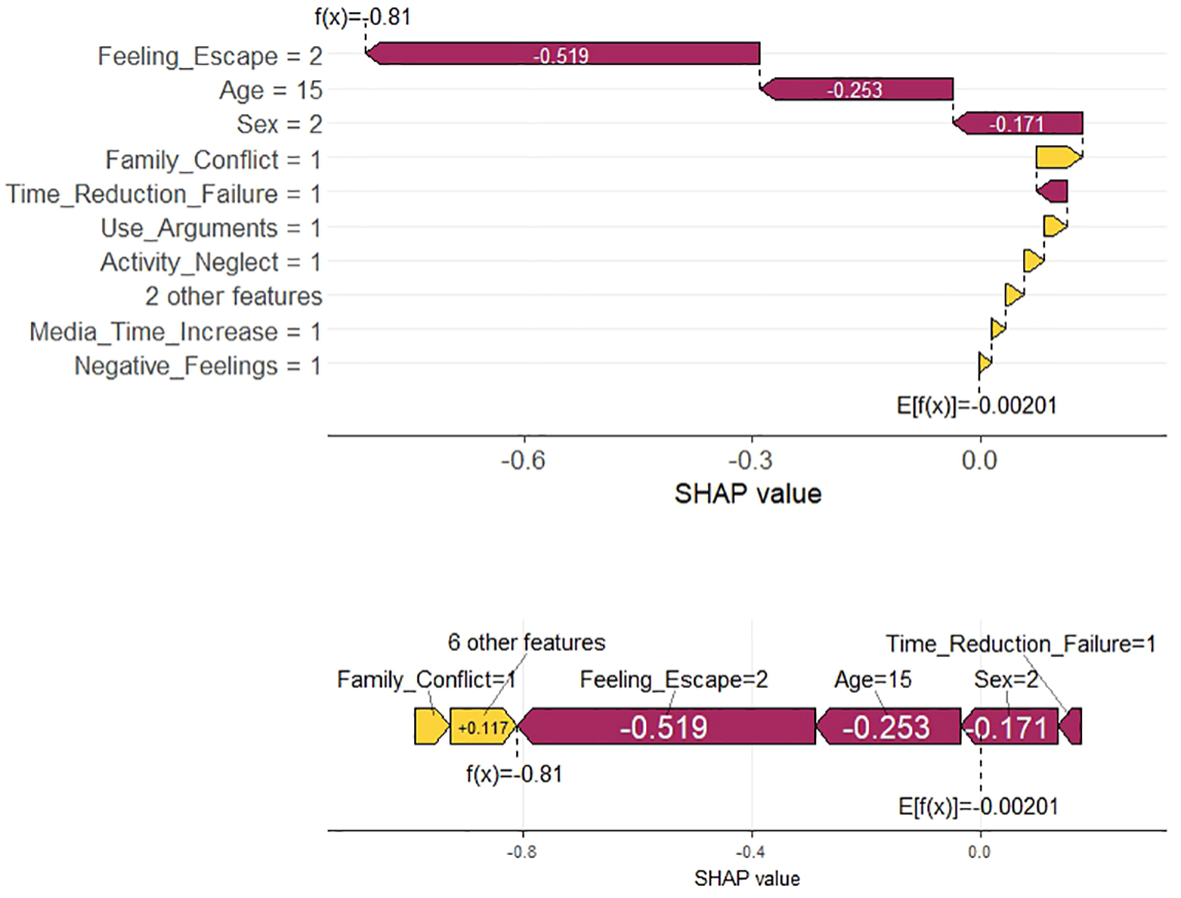
The predictive role of features for specific samples.

**Figure 3** is a SHAP level force diagram showing the characteristic contribution of specific samples in model prediction. The starting baseline prediction is 0.00201, and the model’s final prediction is 0.81 after accounting for sample characteristics. Feeling escape (-0.519), age (-0.253), and sex (-0.171) were the characteristics that contributed most strongly to reducing the predicted SWB in this sample. Of all the variables, only the characteristic of time reduction failure had a positive effect on the predicted value. The other variables, to varying degrees, reduced the predicted value of the individual’s SWB.

**Figure 4:**
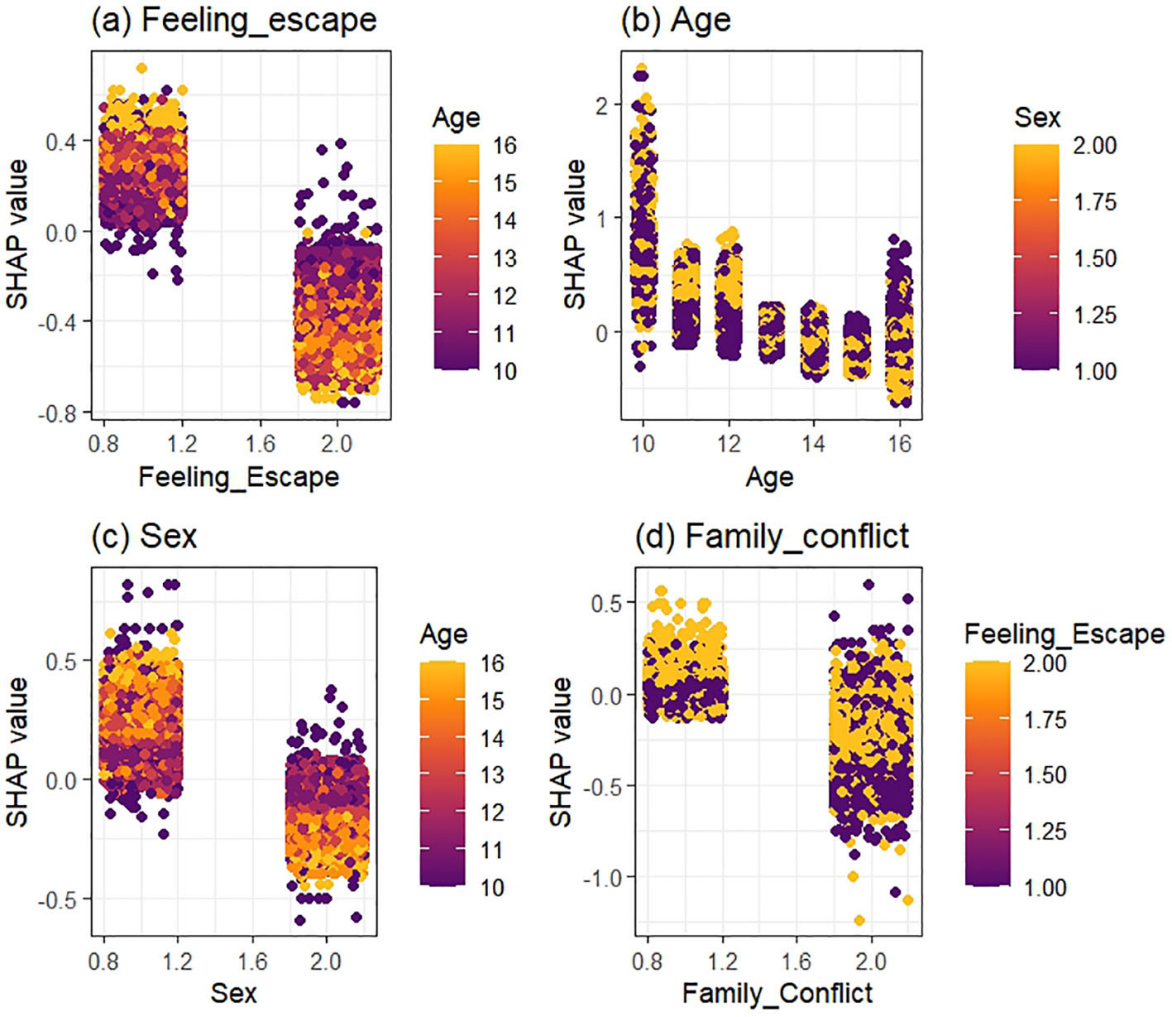
The feature dependence of important factors in the XGBoost model. **(a)** feeling escape; **(b)** age; **(c)** sex; **(d)** family conflict.

**Figure 4** illustrates the interaction between these features. Each subfigure reveals the interrelationship between two features and their joint effect on SHAP values and model predictions. Changes in the horizontal axis and legend represent changes in the feature values. The vertical axis represents the SHAP values of the features.

In **Figure 4a**, this study found that there is a negative effect of feeling escape on SWB. The older age group has a positive effect on the predicted value in absence of feeling escape. There is a negative effect of older age group on predicted values in the presence of feeling escape. From **Figure 2**, it can be seen that increasing age will gradually weaken or even become negative on the predicted value of SWB. **Figure 4b** shows that when the age is small, women contribute more positively to the predicted value. When the age is older, females on the contrary have a negative influence on the predicted value. **Figure 4c** shows that males are more likely to increase the predicted value of SWB. When the sex is male, increasing age makes the positive effect on the predicted value more significant. When the sex is female, increasing age makes the negative effect on the predicted value more significant. **Figure 4d** shows that family conflict may have a negative effect on the predicted values. The combined effect of feeling escape and family conflict can exacerbate the negative effect on the predicted values.

**Figure 5:**
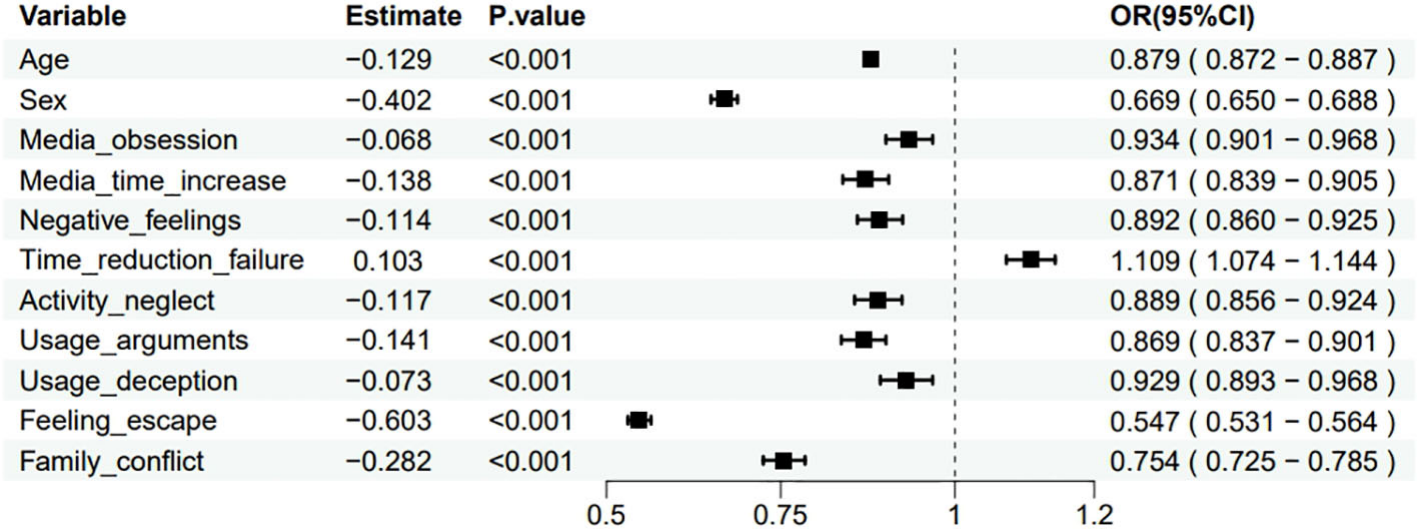
Multivariate logistic regression analysis of variables on SWB.

**Figure 5** presents the results of the multifactor logistic regression analysis. Sociological and demographic variables such as age and sex, as well as problematic social media use variables, were significantly associated with SWB. Age (OR = 0.879, 95% CI: 0.872 - 0.887, *p* < 0.001) was significantly negatively correlated with SWB. This suggests that SWB tends to be lower with increasing age among adolescents. Sex (OR = 0.669, 95% CI: 0.650 - 0.688, *p* < 0.001) was also significantly associated with SWB, with females showing lower levels of SWB compared to males. Among the problematic social media use variables, all indicators were significantly associated with SWB. Media obsession (OR = 0.934, 95% CI: 0.901 - 0.968, *p* < 0.001), media time increase (OR = 0.871, 95% CI: 0.839 - 0.905, *p* < 0.001), negative feelings (OR = 0.892, 95% CI: 0.860 - 0.925, *p* < 0.001), activity neglect (OR = 0.889, 95% CI: 0.856 - 0.924, *p* < 0.001), usage arguments (OR = 0.869, 95% CI: 0.837 - 0.901, *p* < 0.001), usage deception (OR = 0.929, 95% CI: 0.893 - 0.968, *p* < 0.001), feeling escape (OR = 0.547, 95% CI: 0.531 - 0.564, *p* < 0.001), and family conflict (OR = 0.754, 95% CI: 0.725 - 0.785, *p* < 0.001) were significantly negatively associated with SWB. These results indicate that higher levels of these behaviors or experiences were associated with lower SWB. Time reduction failure (OR = 1.109, 95% CI: 1.074 - 1.144, *p* < 0.001) was the only positively correlated indicator, suggesting a slight positive association with SWB.

## 4 Discussion

Based on machine learning and multiple logistic regression analysis, this study systematically examined the relationship between problematic social media use and SWB among adolescents and addressed the research questions. The results showed that, with the exception of time reduction failure, all dimensions of problematic social media use were negatively associated with adolescents’ SWB. This indicates that problematic social media use is generally closely associated with lower SWB. After accounting for complex nonlinear relationships, the XGBoost model further identified feeling escape, age, sex, and family conflict as core predictors of adolescents’ SWB. This indicates that both problematic social media usage characteristics and demographic factors play significant roles in predicting SWB. Furthermore, the interpretive results from Gain and SHAP suggest that age and sex, together with problematic social media use, form a complex pattern of associations Multivariate logistic regression analysis further estimated the direction and strength of the associations between these variables and SWB. Overall, this study revealed the complex structural relationships between adolescents’ SWB and various dimensions of problematic social media use.

### 4.1 Sociological and demographic factors

This study examined the association between sex and age and adolescents’ SWB, and the conclusions reached are consistent with other literature formulations. Age is negatively correlated with SWB (46). Girls tended to have lower SWB than boys (50).

Adolescents’ SWB may be affected by a number of factors as they age. These factors include an increased sense of self and concerns about appearance, body, and relationships brought on by the physical changes of adolescence (51, 52). At the same time, increased academic and achievement pressures may also affect adolescents’ SWB (53). Feelings of inadequacy due to social comparison and stress in family relationships due to increased parental supervision and restrictions may also be influential factors (54). These factors may not only be associated with lower levels of adolescents’ SWB in themselves, but may also further exacerbate declines in SWB through social comparison processes (55). There is a large interaction between age and sex, which together influence adolescents’ SWB. As girls grow older, there are high socio-cultural expectations for girls in terms of appearance, academic achievement, and interpersonal relationships (56). This often brings about additional stress and feelings of dissatisfaction (57), which reduces SWB. In contrast, the situation is different for boys. Although boys also face a certain amount of stress, as they grow older, they are often expected by society to display more confident, independent and risk-taking behaviors (58). These traits may make it easier for them to succeed in academic and social activities, thus enhancing SWB.

### 4.2 Problematic media use factors

This study found that, with the exception of time reduction failure, all other dimensions of problematic social media use were negatively associated with adolescents’ SWB. This indicates a strong consistency in the negative association between problematic social media use and adolescents’ SWB; although the strength of the effect varies across different dimensions, they are generally associated with lower levels of SWB. Since the machine learning results further identified feeling escape and family conflict as the most critical predictors, this paper focuses on discussing these two variables.

This study further validated the negative correlation between feeling escape and SWB (59). This finding is consistent with the compensatory internet use theory discussed earlier. According to this theory, individuals may use the internet as a compensatory means to alleviate negative emotions or real-life stress. Adolescents’ use of social media to escape negative emotions is a concrete manifestation of this avoidance coping mechanism (25).There may be a complex interaction between the two rather than a simple unidirectional causal relationship. Research has shown that adolescents with lower SWB are more likely to escape negative emotions through problematic social media use (60). Although this approach may provide some emotional relief in the short term, it is generally not a healthy coping strategy in the long run (27). As adolescents grow older, they face an increasing number of pressures and problems. Relying on social media to escape negative emotions may prevent them from confronting life’s challenges head-on and effectively resolving issues (2). At the same time, avoidance tendencies may also lead to compulsive social media use, resulting in social media fatigue (61). This chain reaction not only exacerbates emotional issues such as depression and anxiety, but also significantly reduces SWB (62). In contrast, adolescents who do not rely on such unhealthy coping mechanisms are more likely, as they grow older, to learn to face reality and master emotional regulation skills, thereby enhancing their SWB (41).

Our findings support the findings of previous studies that family conflict is negatively related to adolescents’ SWB (63–65). Family conflict undermines family relationships and negatively affects SWB in a number of ways (66). First, family conflict undermines adolescents’ sense of belonging and security and alienates family members from each other (67). Lack of love and support can exacerbate adolescents’ emotional distress, such as feelings of loneliness and anxiety, which can significantly reduce their SWB (68). In addition, family conflicts are often accompanied by negative emotions and communication barriers. This can weaken understanding and trust among family members, further affecting family harmony and reducing SWB (69). At the same time, for adolescents, family conflicts may be one of the key sources of real-life stress that drives them to turn to social media as a means of escaping negative emotions (70). Consequently, when adolescents are confronted with family conflict, they may escape negative emotions through problematic social media use rather than when they are actively problem solving. This behavior may further undermine SWB, creating a vicious cycle.

There may be an interaction between age, sex, feeling escape, and family conflict, and the pattern of this interaction is significantly associated with adolescents’ SWB. This is consistent with previous findings. As adolescents grow older and face more complex academic and interpersonal pressures, they are more likely to use social media as a means of emotional escapism (33). This tendency is particularly pronounced among females, which may be related to their greater emotional sensitivity, tendency toward social comparison, and interpersonal sensitivity, thereby further amplifying the negative impact on SWB (71). At the same time, in situations of family conflict, adolescents are more prone to experiencing negative emotions and tend to adopt avoidance coping strategies, which exacerbates the adverse effects on their SWB.

An unexpected finding was that time reduction failure was positively associated with SWB. However, this result does not necessarily contradict compensatory internet use theory, which primarily emphasizes the use of online activities to cope with negative emotions and psychosocial difficulties (25). In contrast, time reduction failure may reflect difficulties in regulating usage behavior and therefore capture a different dimension of social media use. Given its relatively small effect size and low importance in the predictive model, this finding should be interpreted with caution. One possible explanation is that adolescents who report unsuccessful attempts to reduce their social media use may differ from those who have never attempted to do so. Having attempted to reduce use may reflect some degree of awareness of their online behavior and a willingness to regulate it, which could be associated with better psychological adjustment (72). Further research is needed to examine this possibility.

### 4.3 Strengths and limitations

This study has several strengths. Firstly, the research is based on 2017/18 HBSC research data. The HBSC is an international study designed to collect data on the health behaviors of children and adolescents. The data collection process is standardized and has a large sample size, which ensures the reliability and representativeness of the data. Second, this study combines machine learning methods with multiple logistic regression. It not only identifies the key factors influencing adolescents’ SWB and their relative importance but also examines the statistical associations among these variables. This approach enhances both the predictive power and the interpretability of the results. Furthermore, this study examines the interaction effects among variables. By analyzing these interactions, it provides more nuanced evidence to support an understanding of the complex relationship between social media use and SWB.

However, this study has five limitations. First, the HBSC data are cross-sectional. Estimates derived from these data may be subject to certain biases and are unlikely to support rigorous causal inferences. Therefore, future studies should employ longitudinal or experimental designs to identify causal pathways. Second, self-reported data may introduce recall bias and social desirability bias. Adolescents’ reports of social media use and related experiences may be overestimated or underestimated, thereby affecting measurement accuracy and, in turn, the estimation results. Third, the dichotomization of key variables constitutes a methodological limitation. While converting SWB from a continuous variable to a binary outcome facilitates classification modeling and policy interpretation, it inevitably results in some information loss and may reduce measurement precision, potentially introducing additional measurement error into the analyses. Similarly, the dichotomization of problematic social media use simplifies its complex behavioral characteristics and may obscure differences in usage intensity and frequency. Finally, although machine learning methods such as XGBoost demonstrate strong predictive performance and can capture complex nonlinear relationships, their interpretability remains limited. While the SHAP method enhances model transparency to some extent, it reflects the contribution of variables to the predicted outcomes rather than causal effects. Therefore, caution is warranted when interpreting these results. At the same time, the “black box” nature of machine learning models limits their direct application in theoretical validation and policy implementation. Furthermore, it should be noted that the data used in this study is not from the most recent survey cycle, as the 2021/22 HBSC international data remains confidential until October 2026. Future research can update the analysis once the data is made public to validate and extend the existing findings.

### 4.4 Public policy implications

The findings of this study offer more targeted insights for public health policy. The research indicates that social media use characterized by feeling escape is a significant predictor of adolescents’ SWB. This suggests that intervention efforts should not be limited to controlling screen time but should instead focus on enhancing emotional regulation skills. For example, school-based mental health education and emotional management training can help reduce avoidant dependence on social media. At the same time, family conflict plays a significant predictive role and interacts with feeling escape, indicating that problematic use is embedded within the context of family relationships. Therefore, it is necessary to expand interventions from the individual level to the family level. By improving parent-child communication and the family support environment, related risks can be mitigated at their source. Furthermore, differences exist across age and sex groups regarding SWB and its underlying mechanisms. Older adolescents and females are particularly vulnerable, implying that policy design should prioritize stratified and targeted interventions. Overall, relying solely on platform regulation is unlikely to address the core of the issue. Moving forward, a balance must be struck between external constraints and internal capacity-building by integrating platform governance, mental health promotion, and family support to establish a systematic intervention framework.

## 5 Conclusion

This study, based on HBSC data and utilizing XGBoost models combined with multiple logistic regression analysis, examined the relationship between problematic social media use and SWB among adolescents. The results indicated that, with the exception of time reduction failure, all other dimensions of problematic social media use were negatively associated with SWB. A sense of feeling escape, family conflict, age, and sex are key predictors of adolescents’ SWB, and there are complex interactions among these factors. The findings suggest that interventions should not focus solely on the duration of social media use but should also emphasize adolescents’ emotional regulation skills, the quality of family communication, and targeted support for high-risk groups. Future research could adopt a longitudinal design to further explore the causal relationship between problematic social media use and SWB, as well as its underlying mechanisms.

## Data Availability

The original contributions presented in the study are included in the article/**Supplementary Material**. Further inquiries can be directed to the corresponding author.
